# A Model for Good Governance of Healthcare Technology Management in the Public Sector: Learning from Evidence-Informed Policy Development and Implementation in Benin

**DOI:** 10.1371/journal.pone.0168842

**Published:** 2017-01-05

**Authors:** P. Th. Houngbo, H. L. S. Coleman, M. Zweekhorst, Tj. De Cock Buning, D. Medenou, J. F. G. Bunders

**Affiliations:** 1 Ministry of Health, Cotonou, Republic of Benin; 2 Polytechnic School, University of Abomey-Calavi, Abomey Calavi, Republic of Benin; 3 Athena Institute, Vrije Universiteit Amsterdam, Amsterdam, The Netherlands; University of Texas at San Antonio, UNITED STATES

## Abstract

Good governance (GG) is an important concept that has evolved as a set of normative principles for low- and middle-income countries (LMICs) to strengthen the functional capacity of their public bodies, and as a conditional prerequisite to receive donor funding. Although much is written on good governance, very little is known on how to implement it. This paper documents the process of developing a strategy to implement a GG model for Health Technology Management (HTM) in the public health sector, based on lessons learned from twenty years of experience in policy development and implementation in Benin. The model comprises six phases: (i) *preparatory analysis*, assessing the effects of previous policies and characterizing the HTM system; (ii) *stakeholder identification and problem analysis*, making explicit the perceptions of problems by a diverse range of actors, and assessing their ability to solve these problems; (iii) *shared analysis and visioning*, delineating the root causes of problems and hypothesizing solutions; (iv) *development of policy instruments for pilot testing*, based on quick-win solutions to understand the system’s responses to change; (v) *policy development and validation*, translating the consensus solutions identified by stakeholders into a policy; and (vi) *policy implementation and evaluation*, implementing the policy through a cycle of planning, action, observation and reflection. The policy development process can be characterized as bottom-up, with a central focus on the participation of diverse stakeholders groups. Interactive and analytical tools of action research were used to integrate knowledge amongst actor groups, identify consensus solutions and develop the policy in a way that satisfies criteria of GG. This model could be useful for other LMICs where resources are constrained and the majority of healthcare technologies are imported.

## 1. Introduction

Problems with governance in low- and middle-income countries (LMICs) can lead to ineffective and conditional donor aid, the waste of scarce resources and societal disillusionment with public institutions. Moreover, it leads to a lack of available resources for, and accessibility to, health care, which affects the poor and marginalized most [1]. GG in the health sector, especially in healthcare technology management (HTM), looks like “moving a mountain” for many LMICs where a great number of contextual challenges exist. Since about 95% of the healthcare technology used in these countries is imported [2], numerable layers of bureaucracy are present, each being prone to corruption and administrative malfeasance. Furthermore, imported technology is often not tailored to the specific medical and technological demands of the receiving nation, leading to further problems of outreach and public aid [3–5]. Ineffective HTM has been reported in many poor countries in sub-Saharan Africa [4, 6–8]. The aim of this study is to analyze these problems and intervene with environment-specific solutions, through the concept of “good governance” (GG). This research attempts to explore how weak governance in the public health sector can be transformed into strong governance, and what that process looks like.

The GG concept has evolved, since it was first discussed at the World Bank’s Annual Conference on Development Economics in 1991 [9], into a set of principles and conditional prerequisites for LMICs to strengthen the functional capacity of their public bodies, and receive donor funding [10–12]. Although ample attention has been paid to the concepts constituting GG [13–16] as well as general GG recommendations, suggestions and advocacies, little has been published on the benefits and pitfalls of implementing GG practices. Few evaluation studies exist on practices to transform a poorly performing governance system to one that exemplifies the tenets of GG [12, 17–19].

In this article we present a good practice—the HTM policy process in Benin. We describe in detail the step-wise rationale to bridge the implementation gap between general GG principles and contextualized practices, over the past two decades. We will discuss the strength of our approach in bounded rationality [20], as a generalizable model on “how to implement GG principles” in LMICs. Previous research of Houngbo et al. [21] investigated problems in HTM in Benin showing that the sub-sector lacks functional capacity and appears vulnerable to corruption. Additionally, ineffective public procurement (PP) of healthcare technologies has been criticized by the Ministry of Health (MoH) as incapable of converting public money into available and accessible healthcare technologies for improved health outcomes [22–27]. Since 1995, many seminars, workshops and surveys have been organized with diverse stakeholder groups, but they have yet to be translated into a successful policy, such as the first national maintenance policy for healthcare infrastructure, equipment and vehicles of 2002 that was halted due to a lack of political will and budgeted action plan. Piecemeal attempts by the MoH, and isolated, short-term actions of donors have tried to improve HTM capacities, but have not been sufficiently coordinated to deal with problems. HTM in Benin has typically favored top-down policy development and implementation whilst neglecting local experiential knowledge, thus overlooking characteristics of GG [21].

Since 2006, participative action research has been conducted to create environment-specific solutions to the problems in HTM, with a policy development process that satisfies criteria of GG. Principles of participatory action research and GG have been combined to gather insights into the challenges that health facilities in Benin face. Together, these have guided research actions and been enriched by contextual knowledge from the user-level.

The aims of our study were to develop detailed and validated insights into HTM problems at the local, meso- and macro-level, to help design—alongside policy makers, hospital managers, maintenance technicians, donors and users of healthcare technologies—policy actions to progress towards GG, and to reduce the weaknesses in HTM over the period 2006–2015. The guiding research questions were: (i) what would a bottom-up and inclusive implementation strategy to realize GG in HTM look like? (ii) What are the key lessons learned from the application of the developed strategy in Benin?

In this article, we present the principles of the developed GG Implementation (GGI) model, followed by its phases, general aims, the research activities that can be utilized during each phase, and a description of how the policy development process proceeded in Benin.

## 2. Principles of the GGI model

Numerous studies have documented the ineffectiveness of policy reforms in Africa. Actors at lower levels in the health sector, for example health workers, are often those responsible for the implementation of policy. As such, these actors have the ability to limit the implementation of decisions made at higher levels [28, 29]; a study in South Africa showed corrupt actors actively undermined governmental attempts to reform PP [30]. Therefore, although international development organizations often provide functional or task-based descriptions of GG and health system stewardship principles, this does little to describe the actors involved in health systems, their roles and responsibilities and their willingness to fulfill those responsibilities [31]. Principal-agent theory posits that the motivations of agents and principals are often not aligned, and thus low-level actors have the discretion to neglect policy decisions made by those higher up. By strengthening the relationship between policy makers and the agents charged with implementation, the extent of goal divergence could be diminished. This notion has been increasingly discussed in the literature as the perceptions of low-level actors and lay people are being incorporated into policies and development projects more frequently [28, 32–34]. It also provides a mechanism through which many principles of GG can be effected. Accountability, transparency, citizen voice, participation and inclusivity are all objectives that, in part, can be improved by an increased diversity of actors taking part in policy making decisions. The success of this idea as a means to fulfilling GG criteria and for effective policy making has been documented in a large-scale review of countries whose health systems have greatly improved [35].

The Interactive Learning and Action (ILA) approach is a participatory action (or transdisciplinary) research approach. It stimulates knowledge co-creation between societal actors and experts, starting from the premise that relevant stakeholders have different institutional/societal backgrounds and hence, their perceptions on the problem at hand, as well as its solutions, are likely to differ. To realize effective knowledge co-creation, it is crucial to make different stakeholder’s perceptions explicit, and enhance mutual understanding and learning. This process takes place through interactive and analytical tools such as interviews, focus groups, dialogue meetings and argumentation trees. Key principles of the ILA approach include involvement of (end-)users, facilitation of knowledge integration, enhancing coalition building, identifying shared visions, enhancement of trust relations, and an emergent design [36]. These principles and their operationalization have been tested in Zimbabwe, South Africa and Bangladesh [37, 38], used in the context of patient participation for setting health research agendas [39], to develop a constructive technology assessment [40], and in interactive policy making for biotechnologies [41]. The adaptability of this approach stems from its deliberative nature and its space to let the design of research activities emerge from the findings.

Principles of GG and the ILA approach (Table 1) were combined and re-formulated as implementation strategies that are prescriptive in nature but can be enacted via the phased process of the ILA approach (see next section). The implementation strategies are described *ex post*, as many new considerations for policy development emerged as a result of the context in Benin, however they were implicit in the design of research activities. As principles of GG have already served to reduce strategic task uncertainty, the implementation strategies attempt to provide more detailed direction for *good practices for GG*, based on reducing the functional task uncertainty of health system stewards.

**Table 1:** Selected good governance and ILA principles.

Good governance (outcome)	ILA (instrument)
Transparent and enlightened policy making [14]	Enhance the societal and scientific capacity to deal with complex problems through knowledge democratization [36, 42]
Lack of regulatory burden [43]	Understand the perceptions of the problems of different actors [42, 44]
Inclusivity (complete stakeholder involvement) [13, 43–45]	Establish trust relationships [36]
Accountability (self-accountability and government accountability to the public) [13–15, 42–44, 46–48]	Employ a strategy that is built on a shared future vision [36]
Independent judiciary [43, 49, 51]	Identify needs for knowledge, adapt research directions [44]
Freedom of speech [42, 49–52]	Anticipate the risks and benefits of possible interventions [36, 42, 44]
Fighting corruption [42, 47–49, 51]	Knowledge integration and building consensus [42, 44]
Effectiveness and efficiency in processes and institutions [15, 42–45, 47, 48, 50, 52]	Guide and/or coach intermediaries [36]
A strong civil society and citizen voice heard in public affairs [13, 14, 43]	Emergent design [36]
Enhance public-private partnerships [47]	

The five main strategies of the GG implementation (GGI) model are (Table 2): (i) develop evidence-informed and action-oriented knowledge for sustainable development, (ii) employ a strategy that is built on a shared future vision and anticipates the risks and benefits of possible interventions, (iii) engage and facilitate inclusion of the entire collective of relevant stakeholders, (iv) prevent corruption, and (v) work for independent justice. Alongside the strategy to prevent corruption, new sub-strategies are introduced that are relevant to all phases of policy development and the context; informed by experiences with attitudes in the civil service, government and public. These are:

1. “Depoliticizing” public decision-making is based on the aim of appointing the rightly qualified and deserving public servant to the correct position, independently of his affiliation to a political party or social or ethnic group.
2. “Patriotism” can be interpreted as civil servants exhibiting a high-level of concern for the well-being of the country, and respecting, defending and maintaining public goods and services. This philosophy was fundamental to successful HTM reform in Costa Rica [3] through the actions of a dedicated HTM workforce.
3. “Taking swift, punitive action against corruption” stipulates public servants (policy makers, hospital managers, equipment users, technicians and engineers) being held responsible for their administrative misconduct independently of their social or political status. Corrupt attitudes have endured in Benin due to the existence of political impunity. Similar actions need to be taken against corrupt actors outside the public administration, including healthcare technology suppliers.
4. “Civism” can be understood as “good citizenship and discipline”. It means the “priority given by a citizen to its nation or country’s interests before his own interests” [53].
5. “Strengthen capacity in institutions” refers to the adequate training of human resources in HTM. By developing the skills of both high-level public officials and hospital-based health professionals, implementing change within the HTM system would be a much more responsive process.

**Table 2:** The implementation strategies of the GGI model.

**1**	**Develop evidence-informed and action-oriented knowledge for sustainable development**
1.1	Enhance the societal and scientific capacity to deal with complex problems through knowledge democratization
1.2	Stimulate transparent and enlightened policy making
1.3	Integrate knowledge and build consensus
1.4	Enable joint problem formulation between scientific and societal actors
1.5	Moderate excessive regulatory burden
**2**	**Employ a strategy that is built on a shared future vision and looks to anticipate the risks and benefits of possible interventions**
**3**	**Engage and facilitate inclusion of the entire collective of relevant stakeholders**
3.1	Identify needs for knowledge
3.2	Establish trust relationships
3.3	Promote freedom of speech
3.4	Understand the perceptions of the problems of different actors
3.5	Guide and/or coach intermediaries
**4**	**Prevent corruption**
4.1	Drive for effectiveness and efficiency in processes and institutions
4.2	Engage civil society and ensure citizen voice heard in public affairs
4.3	Depoliticize public decision-making
4.4	Promote patriotism (develop a shared vision for national progress and the adequate provision of social services)
4.5	Enhance government accountability to the public and the self-accountability of public servants
4.6	Take swift, punitive action against corruption
4.7	Enhance civism (good citizenship and discipline)
4.8	Enhance public-private partnerships
4.9	Strengthen capacity in institutions
**5**	**Work for independent justice**

## 3. Phases of the GGI model

**Fig 1:**
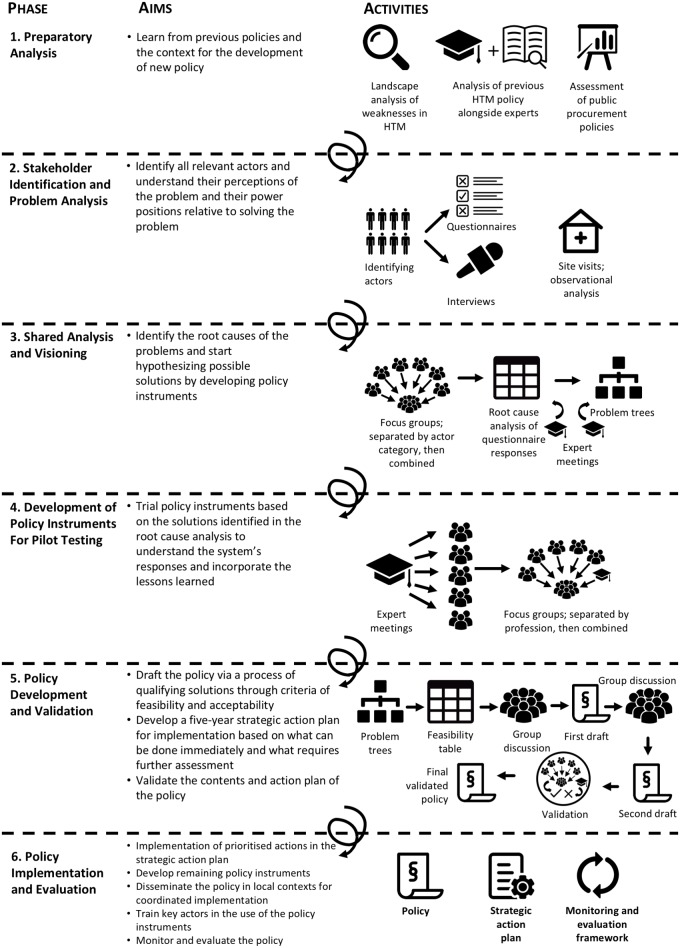
A model for evidence-informed policy making, utilizing the perceptions of state and non-state actors to improve healthcare technology management.

The GGI model is roughly structured along six phases that employ different research and managerial activities in each (Fig 1). This is based on the ILA approach that is organized into five phases: (i) initiation and preparation, (ii) in-depth study of needs and visions, (iii) integration, (iv) priority setting and planning, and (v) project formulation and implementation [36]. In the GGI model, phase 2 of the ILA approach is divided in two, giving rise to six phases. Below, each phase of the GGI model is briefly described and illustrated by its application in HTM policy development in Benin.

### 3.1 Phase 1: Preparatory analysis

The aims of this phase are to learn from the effects of previous policies, assess the background context of HTM and assess the needs for a new policy. Relevant activities include desk research, key informant interviews and workshops to gain an up-to-date understanding of the situation. In Benin, this comprised desk research via three main activities: (i) a preliminary analysis of weaknesses in Benin’s HTM sub-sector [21], (ii) an analysis of Benin’s first HTM policy [24], and (iii) a quantitative analysis of Benin’s first PP codes’ effects on the acquisition prices of healthcare technologies [54]. Each activity is further elaborated, below.

The preliminary analysis comprised two surveys carried out in the six southern departments in Benin during 2006–2007 to:

1. Identify the weaknesses in the HTM system. Data were collected through observational visits, interviews and questionnaires in 11 health centers/hospitals.
2. Determine the extent of disparity between what technologies were documented in each health facility and what was actually available, to facilitate the procurement of essential medical devices for poorly equipped health facilities, and to identify weaknesses in Benin’s HTM cycle. Data were collected through observational visits, document analysis, interviews and questionnaires in 310 health centers/hospitals.

The study was part of a development project commissioned by Coopération Bénin-Union Européene, entitled *Projet 8*^*ème*^ *FED d’Appui au Secteur Santé (08 ACP-BEN 027)*. Semi-structured interviews were conducted with policy makers from the MoH and Ministry of Finance, hospital managers, users of medical equipment, maintenance technicians, some local suppliers, and some international organization representatives, i.e. bilateral or multilateral health development partners. Informed consent was sought in advance of interviews and included in questionnaire forms. The findings highlighted many weaknesses in the HTM system, and particularly drew attention to the high prices paid by government for healthcare technologies. Weaknesses included insufficient human resources to manage equipment and monitor supplier prices, unavailability of spare parts, lack of an annual maintenance budget, and unequal distribution of devices among health care facilities. This suggested PP processes had been hindered by a lack of policy and management tools, such as an up-to-date list of essential medical devices. The preliminary analysis also included a quantitative study of PP that had been specifically maligned by the MoH. The prices of ten medical devices were compared using PP acquisition prices against reference prices quoted to private health providers. The study found that the MoH paid over the odds for medical devices compared to other purchasing bodies, which informed the decision to conduct the in-depth quantitative analysis of the effects of Benin’s PP policies, seen in the third research activity.

**Fig 2:**
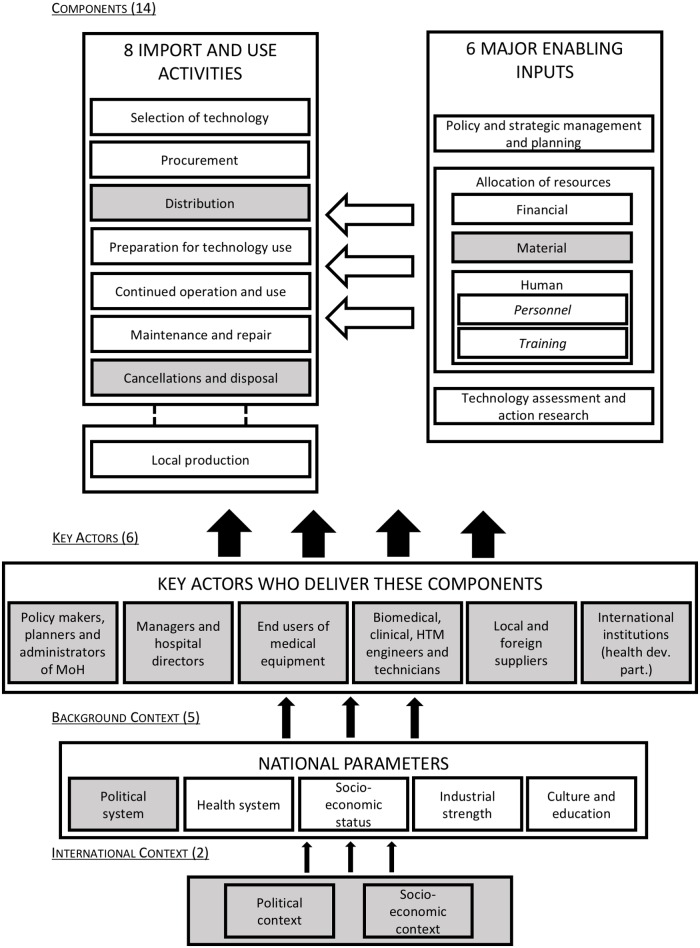
The Temple-Bird Healthcare Technology Package System, tailored to the context of Benin (adapted from [4]).

The second research activity comprised an analytical review of the content of the *“National policy of maintenance of infrastructures*, *equipment and vehicles”* [24] and an assessment of the contextual factors that affected its development and implementation, through discussions with key policy makers in the MoH. These discussions highlighted that a lack of political will and a change in the leadership at the Department of Infrastructure, Equipment and Maintenance (DIEM) responsible for HTM had destabilized efforts for the policy’s implementation. The content analysis showed the policy failed to consider many key components specified in HTM frameworks, such as the Temple-Bird Healthcare Technology Package System (TBHTPS) [4], which includes strategic management and planning, allocation of financial, material and human resources, personnel and training, technology assessment, procurement, distribution, continued operation and use, and maintenance and repair (Fig 2). A failure to consult technical experts and lower-level managers during the policy development process was suggested to underpin the policies ignorance of key components of HTM.

The third research activity analyzed the effects of Benin’s first public procurement code (BPPC) on the public procurement acquisition price (PPAP) of healthcare technologies, via retrospective desk research. The data comprised the PPAPs of devices from 249 PP contracts and the private sector acquisition prices of the same devices, collected from 576 trading sheets from 1995 to 2010. Longitudinal data were organized into interrupted and controlled interrupted time series, taking the implementation of the BPPC and its amendment as intervention points. Segmented linear regression analyses showed that both the first and amended code thoroughly failed to deliver cheaper healthcare technology prices. For the simplest types of equipment (e.g. beakers, splits, X-ray films), a 93% price increase was observed, compared to if the procurement code had never been implemented. To understand this, we looked at what had occurred during the development process. The first BPPC was preceded by a World Bank (WB) assessment in 1999 that stated that the code was plagued by many weaknesses, including: (i) a weak execution body and a lack of institutional capacity at the national level; (ii) a lack of effective policy and management tools; and (iii) a lack of accountability and a highly politicized process of awarding contracts. Due to the delay that would be incurred by revising the code, the government implemented the first BPPC anyway and resolved to review it regularly and amend it as needed. It was also considered that prices may have been increasing due to technological innovations, or conversely, that weaknesses in the PP system made it susceptible to corruption. To disentangle the two, public and private sector acquisition prices were compared for the same devices and the interrupted time series data were controlled for seasonal fluctuations in healthcare technology prices. This showed that the public sector paid significantly more for healthcare technologies than their private counterparts, and that PPAPs were greater than could be accounted for by seasonal fluctuations, suggesting that suppliers were inflating their margins during international tenders. Despite the efforts made to improve the system via the BPPC’s amendment from 2002–2004, PP frameworks still failed to meet international procedural standards and the required guarantees of transparency and integrity [55].

### 3.2 Phase 2: Stakeholder identification and problem analysis

The objective of the second phase is to identify the stakeholders and actor groups to be invited for the policy development process, gain an in-depth understanding of their perceptions of problems in HTM and how their power positions relate to solving these problems. Various data collection methods, both qualitative and quantitative, can be utilized during this phase. Qualitative methods, such as interviews and focus groups (FGs), can help to gain an understanding of the perceptions of state and non-state actors, whereas quantitative methods can be used to provide an empirical analysis of the problem. Research activities in Benin centered on two surveys: (i) questionnaires and interviews with stakeholders to produce an exhaustive list of HTM problems, and (ii) site visits to observe first-hand the causes of ineffective and inefficient use, management and maintenance of healthcare technologies. The results of these research activities have been explored in greater detail elsewhere [56].

As a result of the weaknesses identified in Benin during Phase 1, interviewing all stakeholders relevant to HTM to elucidate an exhaustive list of its problems was a priority. Understanding that expertise and knowledge of different components of HTM are held by different actors across the system, every actor group involved in a capacity with health technologies (apart from patients) was recruited for the first survey: (i) policy makers, planners, and administrators at the MoH; (ii) hospital managers and directors; (iii) end users of medical equipment; (iv) biomedical, clinical, or healthcare technology engineers and technicians; (v) local and foreign suppliers, and; (vi) international organization representatives, i.e. bilateral or multilateral health development partners. Equally, as the weaknesses described in Phase 1 were found in many different areas of HTM, the ability of stakeholders to contribute to solving these problems may be different. Inclusion criteria pertained to the willingness of stakeholders to participate, as well as the aim of establishing a diversity of equally represented perceptions, based on our understanding of historically high- and low-power actors in HTM. Of 5302 key actors in HTM in Benin identified, a theoretical sample size of 244 was calculated based on a sample size calculation with 10% precision rate and 95% confidence level for a finite population [57]. In total, 500 questionnaires were distributed, 377 were returned (75.4%) and 372 were used in the analysis (74.4%). Additionally, 259 semi-structured interviews were conducted in 2011 with informed consent. Questions assessed the aforementioned components of the TBHTPS framework, tailored to the specific context of Benin. Participants were also asked to rank other actor groups, based on their degree of political or administrative power that pertained to solving HTM problems. During the second survey, a calculated sample of 117 healthcare facilities were visited (out of 787 in Benin) in 2012 regarding the placing of equipment and the causes of ineffective use, management and maintenance of equipment. During these visits, notes were taken based on the adapted TBHTPS framework.

The results showed that 85% of actors believed that HTM was failing in all components of the TBHTPS framework. The severity of the problems was rated differently by different stakeholder groups. Biomedical, clinical and HTM engineers and technicians rated the severity of the problems the highest. This was followed by decreasingly severe perceptions from users of equipment, managers and hospital directors, international organization representatives, local and foreign suppliers and, the policy makers, planners and administrators at the MoH, respectively. Questionnaires and interviews also identified the relative problem-solving power of actor groups. Policy makers, planners and administrators at the MoH were collectively regarded as the actor group most empowered to solve HTM problems (acknowledged by 39% of the questionnaire respondents and interviewees). This was followed by international organization representatives (16%), hospital managers and directors (10%), local and foreign suppliers (9%), users of equipment (9%) and finally, biomedical, clinical and healthcare technology engineers and technicians (5%).

### 3.3 Phase 3: Shared analysis and visioning

The goal of the third phase is to identify the root causes of problems in HTM and begin devising possible solutions for policy instrument development. During this phase, interactive learning activities can be organized to stimulate mutual learning between different stakeholders, develop a shared definition of problems and causes, and begin devising possible solutions. This includes interviews, FGs, heterogeneous dialogue meetings and design workshops. In Benin, the research activities comprised: (i) identification of the causes of problems identified in the previous phase, (ii) root cause analysis (RCA), and (iii) organization of problems and solutions into problem trees for prospective policy instrument development. The complete results of the root cause analysis are available in a separate article [58].

During the first research activity, several FGs took place with different stakeholders separately. Subsequently these inputs were combined into a larger discussion that included the users of health technologies, clinical, biomedical and health technology engineers and technicians, hospital managers and directors, and policy makers from the MoH. The FGs yielded a range of causes underlying HTM problems, some of which were more commonly identified by one actor group than another. During FGs, participants were also asked to conceive feasible solutions to the problems in HTM. Integrating knowledge between different groups built a consensus on problems and solutions, developed a shared vision for an improved HTM system, and allowed actors to envision future trajectories of the solutions discussed. This pertains to considering the backlash that might be encountered by alternative policy routes.

The RCA was performed during a session amongst experts, using the organized set of problems and causes formulated during the previous research activity. Main causes identified were the unwillingness and self-interested attitudes of policy makers to engage in HTM problems, and the high degree of politicization influencing public sector decision-making.

For the third research activity, problems and causes discussed during FGs and the RCA were organized into problem trees in an expert meeting. The logic guiding these meetings was to identify ‘low hanging fruit’ in the problem trees, i.e. solutions that could be implemented immediately to mitigate pertinent problems in HTM. The solutions prioritized included the development and implementation of: (i) policy and management tools to guide distribution of healthcare technologies; (ii) reference price lists and an essential equipment list for procuring equipment; (iii) generic specifications of equipment and architectural and technical requirement documents for installation and use; (iv) policy and management tools to guide financial resource allocation on the life-cycle costs (LCC) of equipment; (v) a healthcare equipment and maintenance directorate; (vi) policy and management tools for obsolete equipment; and (vii) a new HTM policy with a budgeted action plan.

### 3.4 Phase 4: Development of policy instruments for pilot testing

The aim of this phase is to develop and trial policy instruments based on the solutions identified in the RCA, in an attempt to understand the system’s responses to change and incorporate the lessons learned. This involves integrating the knowledge of previous phases to begin priority setting and planning. Again, interactive learning activities such as FGs, dialogue meetings and workshops, can be organized to negotiate the content of agendas, as well as questionnaires to assess support for the different solutions identified. The research activities conducted in Benin were: (i) development of the pilot policy instruments, (ii) pre-validation of pilot policy instruments, and (iii) final validation of pilot policy instruments.

The first research activity involved recruiting the services of experts to draft policy instruments for three new Zone Hospitals, funded by the African Development Bank and Islamic Development Bank. The policy instruments were developed from solutions devised during the previous phase that satisfied criteria of feasibility and acceptability and could be implemented immediately. An overview of these is presented in Table 3.

**Table 3:** An overview of the policy instruments developed in response to problems that could be tackled immediately.

Policy Instrument	Purpose
Essential medical devices list	Guide the rational distribution of health technologies across facilities. This prioritized the equipment to be acquired by hospitals based on the disease burden of Benin, alongside considerations of their required quantity in clinical units
Generic technical specifications of equipment	Direct PP and hospital management decisions. This included the equipment’s intended intervention, the complexity of the technology, how easy it is to maintain, and the availability of spare parts.
Technical and architectural requirements for equipment installation and use	Provide an overview of the architectural, engineering, electrical and hydraulic demands of healthcare technology equipment, as many problems in HTM had been linked to poor technical planning.
Program of hospital infrastructure	Provide a blueprint of total hospital space, as well as a detailed record of each technical and non-technical unit in the hospital. Calculates equipment’s spatial demands for installation and use.
Equipment reference price list	Guide health technology procurement decisions. The high PPAPs of health technologies had been identified as an important problem in Phase 1; the price list looked to subvert the power historically afforded to suppliers during the tendering process through better price monitoring.

For the second research activity, FGs were conducted to validate the first draft of policy instruments. Apart from local and foreign equipment suppliers, every stakeholder group was consulted. After a second validation procedure with the same stakeholder groups and experts, the policy instruments were implemented and used to guide the procurement of equipment for, and construction of, three Zone Hospitals in Benin (Djidja, Cove, and Djougou). Although the long-term effects of the policy instruments cannot yet be determined, contracts for the basket of medical equipment required by one hospital were reduced by 18–25% [59]. Evaluating the successes and failures of the policy instruments’ implementation is ongoing but will inform implementation decisions for the prospective HTM policy.

### 3.5 Phase 5: Policy development and validation

The policy development and validation phase aims to draft the policy based on a combination of feasible and acceptable solutions. This includes the development of a strategic five-year action plan to guide implementation and which, alongside the contents of the policy, should be validated. Maintaining engagement with stakeholders is key to foster support for the policy and its implementation. Knowledge co-production, through FGs, workshops and expert meetings, can be used to draft the policy, with particular attention to its implementation. The research activities in Benin were (i) to draft the policy and action plan, and (ii) validate the policy.

**Fig 3:**
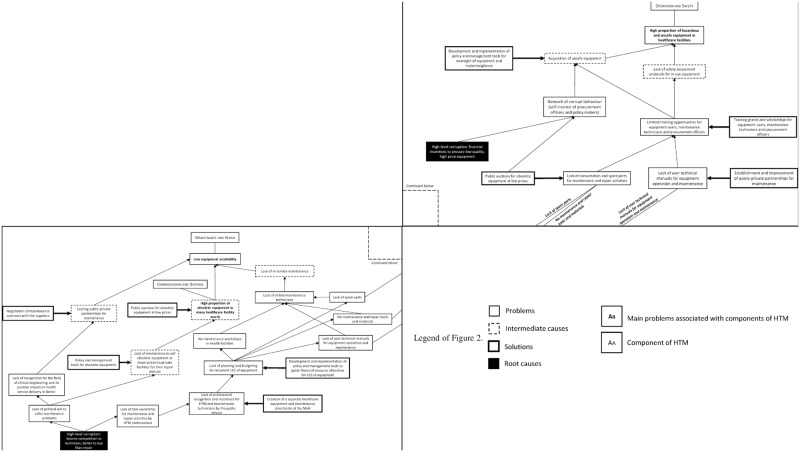
Organisation of problems, causes and solutions relating to the HTM components operation and safety (top right panel), maintenance and repair, and commissioning and disposal (bottom left panel) Also used in [58].

The policy was drafted from solutions mapped in problem trees during phase 3. These solutions were organized into feasibility tables to determine their order of implementation. Fig 3 presents one of three problem trees, which shows the relationships between problems, causes and possible solutions for three key components of HTM: operation and safety, maintenance and repair, and commissioning and disposal. Nine of the fifteen intermediate causes shown in Fig 3 were selected based on their candidacy for feasible resolution: (i) acquisition of unsafe equipment, (ii) limited training opportunities for equipment users, maintenance technicians and procurement officers, (iii) lack of consumables and spare parts for maintenance and repair activities, (iv) lack of user technical manuals for equipment operation and maintenance, (v) high proportion of obsolete equipment in healthcare facility wards, (vi) lacking public-private partnerships for maintenance, (vii) lack of mechanisms to sell obsolete equipment to private healthcare facilities, (viii) lack of planning and budgeting for recurrent LCC of equipment, and (ix) lack of professional recognition and incentives for healthcare technology and maintenance technicians. The main cause of these problems was high-level corruption in HTM processes. Specifically, procuring low quality and expensive equipment was linked to opportunities for financial gain, and a culture of ‘replace over repair’ had emerged from parsimonious attempts to conserve the costs of paying a technician’s income.

Solutions for the above intermediate causes were parsed through criteria of feasibility (availability of competencies and funds) and acceptability (anticipated conflicts of interest between actors) during expert meetings. The feasibility table (Table 4) shows solutions from Fig 3, their typology (health policy-, money-, technical capacity-based) and the length of time for which the solution can provide adequate system improvement (short-, middle- or long-term). Eight appropriate solutions were devised in order of their priority for implementation: (i) development and implementation of policy and management tools for the oversight of equipment and materiovigilance; (ii) training grants and scholarships for equipment users, maintenance technicians and procurement officers; (iii) organization of public auctions to sell obsolete equipment (due to lacking spare parts and repair capacities); (iv) establishment and improvement of public-private partnerships for maintenance; (v) development and implementation of policy and management tools to guide financial resource allocation for LCC of equipment; (vi) creation of a healthcare equipment and maintenance directorate within the MoH, alongside a specific maintenance program; (vii) negotiation of maintenance contracts with suppliers for procured equipment, and; (viii) policy and management tools for obsolete equipment. This process was used for each of the problem trees (constructed from the RCA) when drafting the policy. The policy is unique as the first in Benin to incorporate the use of a conceptual framework and to account policy interventions against a budgeted action plan. The latter is particularly important for LMICs where some interventions depend on the technical or financial assistance of development partners.

**Table 4:** Feasibility table of solutions devised for problems in HTM relating to operation and safety, maintenance and repair, and commissioning and disposal. Also used in [58].

Main Problems	Contributing Causes	Solutions	Priority and Feasibility Assessment		Policy Statements
**High proportion of hazardous and unsafe equipment in healthcare facilities**	1. Acquisition of unsafe equipment	Development and implementation of policy and management tools for the oversight of equipment and materiovigilance	**1**	Money- and technical capacity-based solution Middle-term solution	The MoH has committed to guarantee the permanent and safe availability of equipment and to undertake regular equipment performance assessments
	2. Network of corrupt behaviours (self-interest of procurement officers and policy makers)				
	3. Limited training opportunities for equipment users, maintenance technicians and procurement officers	Training grants and scholarships for equipment users, maintenance technicians and procurement officers	**2**	Money-based solution Short-, middle- or long-term solution Technical and financial development partners willing to support	The MoH has committed to have qualified, motivated and a sufficient number of technical human resources for effective maintenance and management of medical devices
	4. High-level corruption: financial incentives to procure low quality, high price equipment				
	5. High-level corruption: income competition to technician, better to buy than repair				
**High proportion of hazardous and unsafe equipment in healthcare facilities due to lack of oversight**	1. Lack of safety assessment protocols for in-use equipment				
	2. Limited training opportunities for equipment users, maintenance technicians and procurement officers	Training grants and scholarships for equipment users, maintenance technicians and procurement officers	**2**	Money-based solution Short-, middle- or long-term solution Technical and financial development partners willing to support	The MoH has committed to have qualified, motivated and a sufficient number of technical human resources for effective maintenance and management of healthcare equipment
	3. Lack of consumables and spare parts for maintenance and repair activities	Public auctions for obsolete equipment at low prices	**3**	Health policy-based solution Short- or middle-term solution	The MoH has committed to ensure the effective decommissioning, cancellation and disposal of healthcare equipment
	4. Lack of user technical manuals for equipment operation and maintenance	Establishment and improvement of public-private partnerships for maintenance	**4**	Health policy-based solution Short- or middle-term solution	The MoH has committed to guarantee and ensure the preventative and corrective maintenance of equipment
	5. Lack of planning and budgeting for recurrent LCC of equipment	Development and implementation of policy and management tools to guide financial resource allocation for LCC of equipment	**5**	Money- and technical capacity-based solution Short-term solution Requires political support	The MoH has committed to strengthen the transparency of procurement processes for new equipment and to regulate the donation processes of refurbished equipment
	6. Lack of professional recognition and incentives for HTM and maintenance technicians by the public service	Creation of a separate healthcare equipment and maintenance directorate at the MoH	**6**	Money-based solution Short- or middle-term solution Technical and financial development partners willing to support	The MoH has committed to promote the good governance of all components of healthcare equipment management and maintenance
	7. Lack of task ownership for maintenance and repair activities by HTM professionals				
	8. High-level corruption: income competition to technicians, better to buy than repair				
**Lacking public-private partnerships for maintenance**	1. Lack of recognition for the field of clinical engineering and its positive impact on health service delivery in Benin	Negotiation of maintenance contracts with suppliers	**7**	Health policy-based solution Short- or middle-term solution	The MoH has committed to guarantee and ensure the preventative and corrective maintenance of equipment
	2. Lack of political will to solve maintenance problems				
	3. High-level corruption: income competition to technicians, better to buy than repair				
**High proportion of obsolete equipment in many healthcare facility wards**	1. Lack of mechanisms to sell obsolete equipment at lower prices to private facilities for their repair and use	Public auctions for obsolete equipment at low prices	**3**	Health policy-based solution Short- or middle-term solution	The MoH has committed to ensure the effective decommissioning, cancellation and disposal of healthcare equipment
	2. Lack of political will to solve maintenance problems				
	3. High-level corruption: income competition to technicians, better to buy than repair	Policy and management tools for obsolete equipment	**8**	Health policy-based solution Short- or middle-term solution	
**Lack of in-service maintenance**	1. Lack of maintenance technicians and training facilities	Creation of a separate healthcare equipment and maintenance directorate at the MoH	**6**	Money-based solution Short- or middle-term solution Technical and financial development partners willing to support	The MoH has committed to promote the good governance of all components of healthcare equipment management and maintenance
	2. No maintenance and repair tools and materials				
	3. No maintenance workshops in health facilities				
	4. Lack of user technical manuals for maintenance and repair				
	5. High-level corruption: income competition to technicians, better to buy than repair				

The drafting process was followed by a series of validation workshops during which the prioritized actions were debated, with particular attention to their implementation, and to create opportunities for stakeholders to take responsibility for implementation actions. The space for heterogeneous dialogue during the workshop facilitated the process of conflict resolution, specifically the redistribution of some responsibilities and funding were heavily debated.

### 3.6 Phase 6: Policy implementation and evaluation

The aims of the final phase are to successfully implement the prioritized actions in the policy and to organize the capacities and activities for monitoring and evaluation of the policy’s implementation. The phase should proceed through reflexive learning cycles of planning, action, observation and reflection during implementation. Local-level experiments and incremental changes to the policy environment offer opportunities to monitor and evaluate the policy’s trajectory. The managerial activities conducted in Benin comprised: (i) mobilization of public and donor financial resources for implementation of the prioritized actions in the policy; (ii) development of the remaining policy instruments for each component of the HTM cycle; (iii) dissemination of the policy to local departments for coordinated implementation; (iv) creation of a permanent technical committee to define performance indicators and supervise all monitoring and evaluation activities; and (v) supervised training of key actors in the use of the policy instruments. Advocacies and lobbying at the MoH, as well as to donors, is necessary to ensure consistent funding for implementing prioritized action in the policy. Although some actions have already been taken with regards to mobilizing resources, the majority of implementation activities in Benin are forthcoming.

## 4. Discussion

In this section, the lessons learned from the results of each phase are described, the future barriers of implementation of the new policy are analyzed, and the strengths and limitations of using the model are presented. Finally, the applicability of the model in other African countries is discussed.

### 4.1 Lessons learned in each phase of the model

From the results of *phase 1* (preparatory phase), the key lessons relate to the insights gained into the weaknesses of HTM system, particularly finding that the procurement market for health technologies is closed and vulnerable to corruption. Furthermore, the failure to implement the first HTM policy was related to top-down approaches employed. This informed the subsequent development process, particularly with the aim of building the policy from the bottom-up, enhancing transparency and participation, and utilizing an action plan to guide its implementation. Through analyzing the failures of previous HTM and PP policies, it was clear that power positions in HTM are complex, and that a lack of political will had kept some previous policies from being implemented.

The main lesson learned from *phase 2* (stakeholder identification and problem analysis) was that different actors held different perceptions of the problems, e.g. HTM problems prioritized by clinical engineers would not necessarily align with those of MoH policy makers. Furthermore, it was found that the ability of each key actor to solve HTM problems (the degree of political or administrative power they possess) was inverse to their perception of the severity of the problems. This means that actors with low power (such as, engineers and technicians) in the system are highly aware of the problems in HTM but have little capacity to resolve them, whereas individuals in powerful positions have little awareness of the problems but possess the authority to implement solutions. Previous policies directed from the top-down had been controlled by a small number of policy makers, and the opinions of stakeholders with clear perceptions of the problem had never been connected to those with the power to change the system. As a consequence, it was necessary to acknowledge and identify, at each level, the relevant key actors who are important as enablers towards more effective and efficient HTM processes, as their support increases the chances of success. It also holds important implications for subsequent phases with regards to managing conflicting opinions, and ensuring that a small number of views from more powerful participants do not dominate FGs.

From the findings of *phase 3* (shared analysis and visioning), we can learn that solutions differ in feasibility. The conditions that must be aligned to realize the aims of certain policy interventions require different combinations of resources, time and contingent factors. For example, during this phase it was possible to identify *quick win* policy instruments that did not require other contingent factors to be present to be implemented, whereas some policy interventions can be better planned for implementation in the future. Another key finding relates to the large number of politicized decisions that influence HTM processes. In some instances, technical decisions had been compromised due to political influence, e.g. politicized health technology distribution has had an effect on the relative accessibility of health technologies across the country. We recognized that the design of the new policy development process should be structured to limit the opportunities for corruption. As such, development processes should be transparent, and the decisions set forth in policy documents should be evidenced by technical knowledge produced during interactive research activities.

There were three lessons learnt from *phase 4* (development of policy instruments for pilot testing):

1. *Strong resistance*: One of the key challenges was satisfying (or in some cases, overcoming) the combination of official and unofficial interests of different actors during policy instrument development. Their implementation, especially the *architectural and technical requirements for equipment* document, faced strong resistance as it acted against the interests of infrastructure contractors and equipment suppliers. Civil engineers strongly disapproved as more efficient construction of infrastructure projects would decrease the amount of future reparative works they could solicit. Additionally, the generic technical specifications of equipment worked by ignoring considerations of brand and stimulating international open tender competition, yet was lobbied by many suppliers to have their equipment preferred over others’ for specific types of interventions.
2. *Bottom-up integration of technical knowledge*: Policy decisions can be enriched by using experiential knowledge as it offers, for top-level decision makers who may not have the technical knowledge to solve a problem, a thoroughly discussed solution which is supported by actors charged with implementation. Training high-level government personnel in this approach, alongside committed stakeholders and transparency to diminish the space for closed-shop corruption, can bridge the gap between political decisions and technical knowledge.
3. *Saving scarce resources*: The 25% reduction in procurement costs, eliminated by the equipment reference price list, is a hopeful indication for the future effects of the policy instruments, and supports the idea that previous PP decisions had not been fully executed with the best interests of Beninese society in mind.

The lessons from *phase 5* (policy development and validation) illustrate that changes to the formal structure of the HTM system are necessary to reduce the space for poor practices. The joint development of policy instruments to direct the processes of health technology procurement, distribution, resource allocation and maintenance will begin to change the values and practices of actors. Particularly, the establishment and formalization of an independent medical device directorate will promote the importance of HTM and provide it with the central authority and resources to help it manage HTM processes. By anchoring this control in law, it has already been observed to improve economic efficiency.

### 4.2 Future barriers of implementing the policy

Predictable barriers are those which have some scope for management, such as, stakeholder resistance or the lack of financial, technical, and political support. Financial support requests can be improved if, during the submission of budgeted action plans to donors, policy implementation activities are aligned to those donors who promote a similar remit of activities. To improve technical support, transparency and accountability in government can help rebuild trust between high-level MoH officials and lower-level HTM professionals, and improve the knowledge flow from the bottom-up. The implementation of the policy looks to decrease the levels of corruption and unethical practices, however follow-up by a permanent technical committee should be in place to monitor and evaluate the policy’s implementation. To maintain political support, it is important that the technical permanent committee in charge of follow-up preserves a productive correspondence with political authorities.

Unpredictable barriers that could hinder policy implementation processes are radical shifts in political support and similarly unpredictable events (loss of the policy’s key promoters, political instability, and war). A radical lack of political support could, for example, arise if conflicting opinions between a newly appointed Minister of Health and technical HTM director cannot be resolved. This could cause deliberate action by the government to undermine policy implementation. In this context, motivated or unmotivated replacements of officials in the MoH would have a similarly detrimental effect.

### 4.3 Strengths and limitations of using the model

The policy development process was guided by five general principles: (i) develop evidence-informed and action-oriented knowledge for sustainable development, (ii) employ a strategy that is built on a shared future vision and looks to anticipate the risks and benefits of possible interventions, (iii) inclusivity (complete stakeholder involvement), (iv) preventing corruption, and (v) independent justice; and five context-specific sub-principles: (i) depoliticize public decision-making, (ii) promote patriotism, (iii) punitive actions against corruption (iv) enhance civism, and (v) strengthen capacity in institutions. During the implementation of policy instruments in phase 4, the importance of the new principles (patriotism, civism, decreasing politicized decision-making) to the Beninese context was evidenced. Changes made to the PP process, by the new policy instruments, required a different professional culture and practice of procurement staff, as procurement decisions were now directed by stringent guidelines. This was recognized during a goods contracts reception, as staff agreed that greater shrewdness was needed to acquire the correct goods from suppliers under the new guidelines. This could, in part, underpin the 18–25% reduction observed in PPAPs for constructing the new zone hospitals, believed to be due to less payoffs. Many authors have also stressed the concept of patriotism as a key element of GG for public administrations [60, 61], and the philosophy is well illustrated by a quote from Joe Biden: “Fighting corruption is not just good governance. It's self-defense. It's patriotism” [62]. At a national scale, corruption has been linked to poorer health system performance indicators including the maternal mortality ratio [63]. One example, in Costa Rica, described successful HTM reform due to a product of clear oversight, provided by health authorities during the project period, and a dedicated HTM workforce [3]. This edifying example of public officials taking ownership of actions to improve the system is needed in Benin. The new principles are important as they make explicit what hurdles other African countries can expect to encounter. The access to, and maintained engagement of, key stakeholders during the policy development process was also a crucial factor, which is stressed in principles (ii) and (iii). Initially this proved difficult, particularly with healthcare technology suppliers with a vested interest for maintaining the status quo. Recognizing the importance of each stakeholder’s knowledge and encouraging their contribution aided the process of building trust. This has been stressed, under the concept of ‘sensitivity’, in other participatory projects [64]. Although efforts to realize the effective implementation of these new GG principles is highly recommended, they are nevertheless subject to system and contextual limitations in their achievability.

### 4.4 Applicability of the model in the other African countries

The development of the policy in an evidence-informed fashion required quite some time to complete, as well as compliance and continuing support for the research team’s work. The replication of the model in other African countries would be enhanced when tailored to the specific demands of its context. Furthermore, implementing GG practices will always face resistance as the self-interest of the stakeholders are usually at stake. Chances of successful implementation will be increased when values of the society and state are built upon democracy, solidarity and altruism. The translation to other countries also depends on how contextual barriers can be successfully managed, as the six phases are not equally implementable, e.g. the fourth phase requires long periods of experimentation for pilot projects. It is important to state that, during policy development, the project benefitted from the unique position held by the first author, both as a high-level civil servant of the Beninese government, and as an academic researcher, which allowed the introduction of new approaches to build capacity in government (e.g. by incorporating experiential and technical knowledge in policy development, and using monitoring and evaluation frameworks after policy implementation). Indeed, there are many constraints to using research in public health decision making, including the concentration of power, the extent of health policy centralization and democratization, institutional processes and predominant values, the influence of donors and pressure of wider policy strategies [65], and the division in disciplines between researchers and decision-makers [66]; the latter of which this policy development process has sought to diminish. As a result of this process, the need for stronger capacities in governmental monitoring and evaluation has been observed in Benin, as well as greater capacities for conducting policy-oriented research, that partners with society to attain collective health goals, in low-income countries [67]. We hope this will be taken on board by the West African Health Organization (WAHO), which has a wide policy coordination and harmonization program for its member states. Tools to ensure accountability of public officials are required for implementation and success in other contexts. The case presented here contributes to the increasing body of literature that emphasizes the importance of accountability in public decision-making, particularly for those individuals entrusted with prominent public functions and with access to state accounts and funds [68]. Establishing a positive feedback loop of information between state and society is important for continued progress.
